# *CoNaMad—Cohorte de Nacimiento de Madre de Dios*/Madre de Dios Birth Cohort to Study Effects of in-utero Trace Metals Exposure in the Southern Peruvian Amazon

**DOI:** 10.5334/aogh.3152

**Published:** 2021-07-19

**Authors:** William K. Pan, Caren Weinhouse, Ernesto J. Ortiz, Axel J. Berky, Emma Fixsen, Andres Mallipudi, Beth J. Feingold, Suzy Navio, Nelson A. Rivera, Heileen Hsu-Kim, J. Jaime Miranda

**Affiliations:** 1 Nicholas School of Environment, Duke University, Durham, NC, USA; 2 Duke Global Health Institute, Duke University, Durham, NC, USA; 3 Oregon Institute of Occupational Health Sciences, Oregon Health & Sciences University, Portland, OR, USA; 4 Bellevue Hospital Center/Ronald O. Perelman Department of Emergency Medicine, NYU Grossman School of Medicine, NY, USA; 5 School of Public Health, State University of New York at Albany, Albany, NY, USA; 6 Institute for Health and the Environment, State University of New York at Albany, Albany NY, USA; 7 Direccion Regional de Salud, Madre de Dios, Perú; 8 Pratt School of Engineering, Duke University, Durham, NC, USA; 9 School of Medicine, Universidad Peruana Cayetano Heredia, Lima, Peru; 10 CRONICAS Center of Excellence in Chronic Diseases, Universidad Peruana Cayetano Heredia, Lima, Peru

## Abstract

**Background:**

In-utero exposure to mercury and other trace metals pose a significant threat to child health and development, but exposures and health impacts in artisanal and small-scale gold mining (ASGM) environments are poorly defined.

**Objectives:**

We describe the CONAMAD study design, a prospective birth cohort consisting of multiparous women (18 and over) living in rural and peri-urban Peruvian Amazon communities exposed to ASGM.

**Methods:**

Pregnant women are enrolled from health posts across four zones of Madre de Dios, Peru. Data are collected at enrollment, childbirth, and (planned) 36-48 months. At enrollment, hair samples for mercury assessment, demographic and clinical data are obtained. At birth, we obtain venous and cord blood, placenta, hair, toenails, and saliva.

**Findings:**

Two hundred seventy mothers were enrolled at an average 20 weeks gestational age with no differences in maternal characteristics across zones. Two hundred fifteen mothers were successfully followed at birth. We obtained 214 maternal and cord blood samples, 211 maternal and 212 infant hair samples, 212 placenta samples, 210 infant saliva samples, and 214 infant dried blood spots. Data collected will allow for testing our primary hypotheses of maternal malnutrition modifying ratios of cord:maternal blood total mercury (tHg), cord blood:maternal hair tHg, and infant:maternal hair tHg, and whether chemical mixtures (Hg, Pb, Cd) have synergistic effects on infant neurodevelopment.

**Conclusions:**

CONAMAD is designed to collect and store samples for future processing and hypothesis testing associated with in-utero mercury exposure and child development. We have completed the exposure assessments and will conduct a follow-up of mothers to evaluate early child development outcomes, including developmental delay and growth. These data offer insights into disease mechanisms, exposure prevention, and policy guidance for countries where ASGM is prevalent.

## I. Introduction

Methylmercury (MeHg) exposure from dietary intake is likely to lead to detrimental internal doses in susceptible groups, particularly in developing fetuses due to its ability to cross the blood brain barrier [1,2]. When entering the bloodstream, organic mercury (i.e., MeHg) binds to sulfhydryl groups and is distributed throughout the body. MeHg deposited into the brain can become trapped as it slowly demethylates to inorganic mercury that cannot penetrate the blood-brain barrier. The mechanism through which mercury induces neurotoxicity is not clear [3], but many have been proposed, including a glutathione pathway [4], glutamate excitotoxicity [5,6], and mitochondria and reactive oxygen species [7,8]. A key question is related to MeHg toxicity in-utero as higher levels of MeHg exposure may be experienced by fetuses compared to adults in similar environments, and equivalent levels of exposure may lead to greater negative health impacts for individuals exposed to high and/or chronic MeHg in-utero. Cord blood mercury levels are often higher than levels in maternal blood, perhaps due to higher levels of cord blood hemoglobin, which binds free MeHg, increasing active transport of mercury to the fetus [9,10,11,12] or decreasing clearance due to low levels of glutathione (GSH), which binds MeHg and targets it for removal [13]. In addition, the MeHg-cysteine complex is believed to mimic a crucial amino acid, methionine, which a developing fetus may preferentially absorb in the context of maternal undernutrition [14,15]. Maternal malnutrition can exacerbate the impact of chemical exposures on pregnancy complications, such as gestational hypertension or preeclampsia, by increasing maternal MeHg absorption and subsequent mercury transport to the fetus. Alternatively, chemical exposure can impair nutrient absorption, such as protein synthesis or metabolism of zinc (Zn) and iron (Fe) [16]. Unfortunately, epidemiological studies to elucidate the relationship between malnutrition and MeHg are scant.

Neurobehavioral and immune function can also be impaired from perinatal exposure to MeHg with other potentially toxic trace elements (lead [Pb], arsenic [As] and cadmium [Cd]), as compared to those exposed in adulthood [17], possibly due to dysregulation of the epigenome during critical periods of development. Regardless of these toxicological risks, impacts of MeHg and mixtures with MeHg have not been fully evaluated, especially in the context of other known risk factors for child health in a developing region like the Peruvian Amazon. Several studies conducted in the US have reported epigenome-wide alterations in infants exposed perinatally to mercury [18,19], although the gestational window of greatest susceptibility is still unknown. Prior studies report epigenomic deregulation in offspring following maternal malnutrition at the time of conception, but not in the third trimester [20,21,22]. Few comparable studies have been conducted outside of the U.S., and none in Peru or in an artisanal and small-scale gold-mining (ASGM) region.

**Figure 1:**
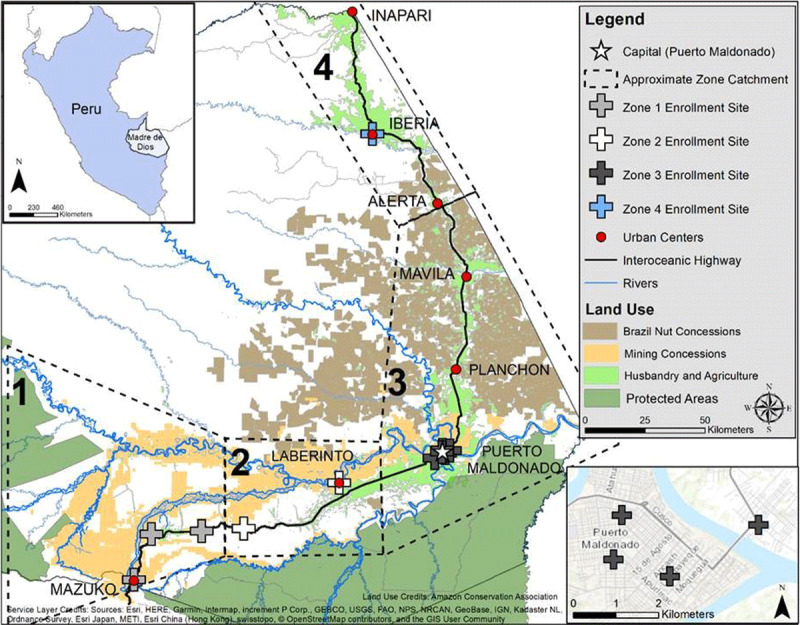
CONAMAD Study Site and Enrollment Locations, Madre de Dios, Peru. Dotted lines represent estimated catchment area; large “+” symbols represent enrollment sites (health centers). ASGM is currently concentrated in the Colorado, Puquiri, Inambari and Malinowski rivers. Sample size obtained in each zone at enrollment and followed at birth: Zone 1, 43 enrolled, 37 births; Zone 2, 67 enrolled, 50 births; Zone 3, 147 enrolled, 121 births; Zone 4, 13 enrolled, 7 births.

In November 2016, we initiated a birth cohort to enable assessment of peri-conceptional, late gestation and early life stages as potential critical windows of developmental exposure to MeHg. The study is in Madre de Dios, Peru, a southwestern region of the Amazon (***Figure 1***). The population of Madre de Dios has high exposure incidence to dietary mercury due to ASGM-related mercury pollution and consumption of high trophic level fish [23,24,25]. In addition, there is high prevalence of malnutrition among women of child-bearing age compared to other regions where developmental MeHg exposure effects were studied, such as in the Seychelles [26,27] and Faroe Islands [28]. The study is named CONAMAD, or *Cohorte de Nacimiento de Madre de Dios* (Birth Cohort of Madre de Dios), which roughly translates as “with mothers” in Spanish. NIEHS (1R21ES026960) provided the primary funding for the design and data collection for this study. Funding for data collection was also provided by the Doris Duke Clinical International Research Fellowship and the Duke Center for Latin American and Caribbean Studies. Additional funding for data processing was provided by the NIEHS Superfund Research Program (P42ES010356) and the Josiah Charles Trent Memorial Foundation Endowment Fund (Grant #17–23). The 36–48-month follow-up is funded by the State University of New York-Albany. The study was motivated by the 2016 El Niño event for which we hypothesized that large-scale flooding from El Niño would increase mercury methylation and bioaccumulation, resulting in higher dietary mercury exposure. We specified three hypotheses:

- Internal MeHg dose of infants in utero as measured in cord blood samples will exceed internal doses as measured by maternal hair Hg levels and this relationship would be mediated by maternal nutrition;
- Poor maternal nutrition will correlate with a higher cord:maternal blood Hg ratio; and
- Exposure to chemical mixtures with MeHg in-utero (in mothers) will lead to greater than additive impacts on infant neurocognitive function via indirect (maternal exposure ➔ gestational hypertension ➔ small for gestational age ➔ cognitive deficits) and direct effects (infant exposure ➔ cognitive deficits).

Funding was used to maximize data collected from mothers and newborns and store the majority of data for future analysis. Data collected include: hair, toenails, blood and placenta for assessment of multi-panel trace elements exposure; clinical health outcomes (hypertension, small for gestational age, child growth, anemia, arbovirus exposure); saliva and dried blood spots (DBS) for epigenetic analysis from newborn and their siblings from the same biological parents (as a control); neurocognitive impairment (developmental delay); and socio-demographic and nutritional measures from survey data. Here we describe the study design, enrollment and birth characteristics of mother-child dyads, and initial findings from enrollment and birth data.

## II. Cohort Description

### 1. Setting – Madre de Dios

Madre de Dios (MDD) borders Brazil and Bolivia (***Figure 1***), and has the lowest population density in Peru (2017 estimated population 141,070, ~20% indigenous [29]) and one of the largest concentrations of biodiversity in the world, with four protected parks and ~15% of Peru’s forest areas [30]. Families living in MDD are involved in a variety of occupations: agriculture, fishing, logging, Brazil nut (castaña) collection, gold mining, construction, and tourism. Houses are modest, made of simple wood or concrete block construction with palm thatch or corrugated zinc roofs, and many lack indoor plumbing and access to electricity from a centralized power network (note: most mothers living in Puerto Maldonado have running water and electricity). Medical care for the study region is available at the Regional Ministry of Health (DIRESA) health posts, which have variable quality and supplies to provide basic medical care. For serious or prolonged illnesses, patients are usually referred to a *Centro de Salud* or one of the two regional hospitals (Hospital San Martin de Porres in Iberia, Hospital Santa Rosa in Puerto Maldonado).

Like many regions around the world, MDD is undergoing rapid development. The construction of the Interoceanic Highway (IOH, completed in 2011) [31,32] and expansion of ASGM over the past two decades, has driven urbanization, internal migration, deforestation, social conflicts and the emergence of dual burdens of disease [33,34,35,36,37]. ASGM is also the underlying cause for widespread MeHg exposure risk as it is related to rapid deforestation, environmental mercury (Hg) release, and biomagnification of MeHg in local fish species and food sources [24,38,39,40,41,42,43]. Our team led the first population-based assessment of mercury exposure in the region, estimating that 58% of the population live in communities affected by ASGM and 43% of women of child-bearing age (WCBA, 15–49 years) have hair mercury levels exceeding 2.2 ug/g, a level corresponding to the WHO provisional tolerable weekly intake [24], which was consistent with estimates from our study of mercury exposure of WCBA in native and non-native communities surrounding the Amarakaeri Reserve [44].

### 2. Characteristics of Women of Child-Bearing Age and Pregnancy in Madre de Dios

In Peru, the Ministry of Health recommends a minimum of 6 antenatal care visits. Clinical care during a prenatal visit usually includes administration (or prescription) of iron tablets or syrup, medicine for intestinal parasites, maternal weight, blood pressure, blood sample (for anemia assessment), belly width, and neonatal tetanus shot, among others. Women usually give births in designated birthing clinics (in MDD: Hospital Santa Rosa and Centro de Salud Nuevo Milenio in Puerto Maldonado, and Hospital San Martin de Porres in Iberia). Other *Centros de Salud* can attend “imminent” births. If a woman arrives at a birthing clinic but is not near initiating labor, she must return home, stay with nearby family, or pay for subsidized housing, which tend to be in disrepair. Thus, it is not uncommon for women, with a controlled, normal pregnancy, to wait until the last minute for an “imminent” delivery that can be attended in her local health facility.

We leveraged two two-stage cluster samples to design the birth cohort: the 2014 Encuesta Demografica y de Salud Familiar (ENDES, Demographic and Health Survey [45]); and our team’s 2014 Interoceanic Highway Study (*Investigacion de Migracion, Ambiente y Salud* or *IMAS*) [24,46]. ENDES sampled 138 households, 112 WCBA and included Puerto Maldonado; IMAS sampled 310 households, 232 WCBA and excluded Puerto Maldonado.

ENDES and *IMAS* exhibited agreement for most indicators of interest. The total fertility rate was approximately 3 births per woman, 12% of WCBA were married (~55% in a consensual union), and WCBA received between 8 and 9 years of schooling. Anemia prevalence among WCBA was 29.1% in ENDES versus 43% in IMAS (identical methods used). Surveys reported WCBA with similar prevalence of short stature (<145 cm, 8–11%), an indicator of increased risk for adverse maternal and child health outcomes, and for prevalence of overweight or obese (body mass index or BMI > 25), which was 66% of WCBA. Both surveys indicated high prenatal and childbirth health care utilization, with 93% of pregnant women from both surveys reporting at least 4 prenatal care visits with the median gestational age of the first visit occurring at three months. Overall, 96% of women from ENDES and 87% from IMAS who gave birth in the five years prior to the survey did so in a health clinic or hospital, and 97% (ENDES) and 87% (IMAS) of births were attended by a health professional (doctor, nurse, or obstetrician). Additional data in ENDES not reported in IMAS included maternal report of underweight at birth (under 2500 grams, 4.3%), very small size at birth (1.5%) and smaller size than average at birth (21%). Both surveys indicated an approximate stunting prevalence around 10% in children under five; however, ENDES reported 51% of children under five are anemic, while IMAS estimated 70%.

### 2. Study Design

CONAMAD is a prospective birth cohort to measure in-utero (fetal) mercury exposure in pregnant mothers and the impact of exposure on newborn health and development. Human subjects research approval is through the Universidad Peruana Cayetano-Heredia (UPCH) Comité Institucional de Ética (CIE) para Humanos (SIDISI 66471), the DIRESA Madre de Dios, and the Duke University Office of Research Support via an Inter-Institutional Agreement with UPCH. Follow-up was further approved by the University at Albany Offices of Research Support via Inter-Institutional Agreements with UPCH.

Enrollment occurred in four zones across MDD to capture pregnant women living in four general categories of environmental mercury exposure (***Figure 1***): (1) Huepetuhe vicinity, representing older and larger gold mining areas; (2) La Pampa vicinity, representing more recent and emerging gold mining; (3) the Puerto Maldonado urban and sub-urban vicinity; and (4) Iberia vicinity, representing non-ASGM impacted communities. These four categories were defined from prior data [24,41,42] and we expected, *a priori*, that mercury exposure would be highest in zones 1 and 2, moderate in zone 3 and low in zone 4. We note that the highest concentration of indigenous communities is in zone 1; however, no indigenous mothers were enrolled.

**Figure 2:**
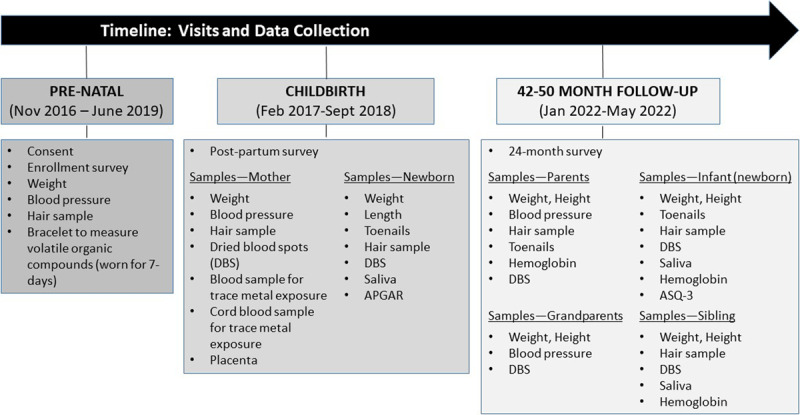
CONAMAD Study Design Overview and timing of data collection. The SARS-COV-2 has delayed the 24-month follow-up by at least 12 months; thus, we will initiate follow-up in January 2022.

The study design involves prenatal enrollment and data collection at birth and 36–48 months of age post-partum, with each contact point involving targeted data collection (***Figure 2***). Pregnant women were enrolled during any trimester prior to 30 weeks gestation. To be eligible, pregnant woman met the following criteria: (1) primary residence in MDD; (2) aged 18–30 years old; (3) reside with a domestic partner or spouse; and (4) have at least one additional child from the same biological parents as the unborn fetus (a child from the same set of parents as a potential control in epigenetic analysis). Exclusion criteria were: (1) clinical diagnosis of type II diabetes before pregnancy; (2) current smoker; (3) residence outside of MDD for more than two weeks during pregnancy (assessed at the time of enrollment); and (4) planning to give birth outside of MDD. Women were invited to participate by study nurses and health professionals at each of the regional hospitals/birth centers or by fieldworkers in close communication with medical personnel. If they agreed to participate, eligibility was ascertained and, if eligible, the interviewer would either review the consent form with the mother or she could review the form herself. If a woman could not read or could not speak Spanish, we provided local translation for her. Consent included approval for individual chemical exposure results to be shared with the local DIRESA and long-term storage of their biological samples for future testing. Once consent was obtained, clinical records of consenting women were stamped to increase the likelihood that a fieldworker would be notified and present at the time of birth. An enrollment survey was administered, a hair sample was obtained to measure prenatal mercury exposure, and a subset of women were given a silicone wristband to wear for seven days to measure exposure to volatile organic compounds (VOCs) and polycyclic aromatic hydrocarbons (PAHs). The enrollment survey includes transcription of prenatal care clinical records, including weight, blood pressure, and supplements administered, diet (frequency of fish consumption by species), and prenatal complications. Previous work in the region by our team on diet helped inform food consumption questions [23,46].

At birth, data collected include: a postnatal survey; maternal and newborn hair samples, newborn toenails, and maternal and cord blood for trace elements exposure assessment (focusing primarily on Hg); placenta; and dried blood spots (DBS) and saliva samples for epigenetic testing (the DBS is also obtained for arbovirus screening). Fieldworkers were able to travel to most birthing centers within an hour, except for Iberia, which is four hours from Puerto Maldonado. Medical personnel at each hospital/birthing center were trained on how to take blood, placenta, hair, and nail samples, in case the fieldworker was not present. Three tufts of hair, from the mother and newborn (if possible) were obtained from the occipital region of the scalp. The hair specimens were cut with stainless steel scissors, secured on self-adhesive note paper with the proximal end fixed in the adhesive part of the paper, and placed in plastic zip lock bags with 2–3 silica gel desiccants. Newborn toenails, if possible, were collected using baby clippers and placed in paper envelope bags. Two 6 ml tubes of maternal blood were obtained using BD Vacutainer® Trace Element Plastic Blood Collection Tubes (with EDTA). Four 6 ml tubes of cord blood were similarly obtained by drawing blood from the already cut umbilical cord after birth. Four placenta punch biopsies were obtained (one punch from each of the four quadrants of the placenta) and stored in cryovials filled with AllProtect® tissue reagent as a stabilizer. Cord blood and placenta samples were obtained in a separate room from the mother to avoid contamination. Saliva was obtained from the newborn using a saliva sponge tip. Dried blood spots from the mother and infant (via heel stick) were obtained using Whatman cards and hemoglobin levels for anemia were evaluated.

Biomarkers we plan to process include total hair Hg, total blood Hg, and other trace elements for maternal and cord blood samples. Banked (stored) samples include: placenta, saliva, DBS, bracelets for VOCs and PAHs. Duplicate and triplicate samples of hair, and blood will also be used for future stable isotope analysis, epigenetic testing, and cortisol.

We initiated a 24-month follow-up in December 2019 with child ages expected to range from 20–33 months, with most visits occurring at approximately 24 months of age. This follow-up was halted due to COVID-19 with plans to re-start the study by January 2022. Data are planned to be collected from the child (newborn), the child’s parents, sibling, and, if present in the household or living nearby, maternal grandparents (***Figure 2***). Anthropometrics, a DBS, and a hemoglobin test will be obtained from all individuals (hemoglobin testing only for the mother, child, and sibling). Hair and toenail samples are obtained from the child and their parents and sibling. Saliva is obtained from the child (if not obtained at birth) and sibling. Blood pressure is obtained from adults (parents and grandparents). Finally, the child will be administered the Ages and Stages Questionnaire (ASQ-3) to screen for developmental delays or disorders (validated up to 60 months) [47], which has been used in many contexts, including in Peru [48,49,50]. Children identified at risk of developmental delay in one of the five areas evaluated in the ASQ-3 will be referred to the Madre de Dios office of community mental health. The follow-up survey includes data on household composition, (individual) dietary consumption (including primary food that is shared with the family and secondary food consumed only by the individual), infant breastfeeding practices, occupation, migration/travel, access to markets and health care, parental stress and parenting, and social vulnerability.

CONAMAD is designed to achieve 80% power with 250 mother-child dyads at the 0.05 significance level to detect a 20% increase in the Hgc:Hgm (c = cord blood; m = maternal blood equivalent from hair) in mothers pregnant during El Niño or whose infants were born having low birthweight compared to mothers pregnant after El Niño (i.e., this assumed a non-El Niño exposure ratio of 1.25 and a 20% loss to follow-up). Unfortunately, funding and IRB approval were not obtained until October 2016, after the El Niño event. Therefore, we re-computed power to detect an increase in the Hgc:Hgm ratio for malnourished mothers compared to non-malnourished mothers, as measured by short stature and/or anemia during pregnancy. Research indicates that iron deficiency, a cause of anemia, can increase maternal absorption of Hg [51]. In addition, research has estimated a 20–30% increase in absorption of MeHg for fetal erythrocytes compared to maternal erythrocytes [52,53]. Therefore, we hypothesize that anemic mothers to have a minimum 20% higher Hgc:Hgm compared to non-anemic mothers, which is the same power calculation as estimated above. Note that a 30% increase in the Hgc:Hgm ratio (consistent with potential increased MeHg in fetal blood from anemia mothers) would have 97% power and, at this effect size, a Bonferroni adjustment with three comparisons still achieves more than 93% power.

### 3. Sample Characteristics at Enrollment

Two hundred seventy pregnant women were enrolled between November 2016 and July 2018 in 12 health posts across the four zones. In 2017, MDD recorded a total of 4137 births [54]; thus, our sample represents approximately 4.4% of all births during the period of study or 7% of births over a one-year period. Just over half of the women were enrolled from zone 3 (surrounding Puerto Maldonado); 41% from zones 1 or 2 (Huepetuhe and La Pampa area, respectively); and 5% from non-ASGM impacted communities (***Table 1***). Women were regularly enrolled over time (Supplemental Figure 1). The average gestational age of enrollment was 20 weeks (***Table 1***), and there were no differences in enrollment timing by zone with 21% of women enrolled during their first trimester, 53% in their second, and 26% in their third (***Table 1***). Other characteristics of mothers at enrollment did not vary significantly by zone, including median maternal age (28 years) and prevalence of short stature (16.6%), with highest prevalence of short stature in mining areas (zones 1 and 2). Median age of male partners was 30 years and most women lived in a consensual union (94%). The most common forms of employment were driving a taxi (19%), farming (16%) and mining (14%). Other types of employment included construction (9%), machine operation and mechanic (8%), and administrative work (7%). We did not record the place of birth for the mother at enrollment; however, for their male partners, 39% were born in Madre de Dios and 35% in Cusco. Overall, 48% of male partners were born in an Amazon region of Peru and 48% in the highlands.

**Table 1:** CONAMAD Enrollment characteristics.


VARIABLE	ZONE					P-VALUE, DIFFERENCE ACROSS ZONE

	1	2	3	4	ALL ZONES	

NUMBER OF MOTHER-CHILD PAIRS (%)	43 (16%)	67 (25%)	147 (54%)	13 (5%)	270	

Median Age, years (mean)	27 (26.9)	28 (27.6)	28 (27.4)	25.5 (25.3)	28 (27.3)	0.2532

Weeks pregnant at enrollment (SD)	20.1 (8.12)	20.7 (7.09)	20.5 (6.53)	16.4 (7.05)	20.3 (6.98)	0.2288

Enrolled in … 1^st^ Trimester (<14 weeks)	27.9%	19.4%	19.1%	38.5%	21.5%	0.1995

… 2^nd^ Trimester (14–26 weeks)	39.5%	50.8%	57.8%	53.9%	53.0%	

… 3^rd^ Trimester (>26 weeks)	32.6%	30.0%	23.1%	7.7%	25.6%	

% Short Stature, ht ≤ 1.45 m	23.30%	20.90%	12.90%	15.40%	16.60%	0.2953

Median age of male partner, years (mean)	31.5 (31.4)	31 (32.2)	30 (30.7)	28 (29.0)	30 (31.1)	0.1859

Mother lives near^1^						

… gold shops	25.6%	20.3%	7.9%	8.3%	13.6%	0.0098

… mining	53.9%	20.3%	7.9%	0.0%	17.7%	<0.0001

… gas station	56.4%	20.3%	37.9%	8.3%	35.2%	0.0050

… road near vehicle traffic	74.4%	25.4%	58.6%	16.7%	51.2%	<0.0001

… trash burning	71.8%	35.6%	37.9%	8.3%	41.2%	0.0007

… farms that burn before planting	51.3%	23.7%	18.0%	50.0%	26.1%	<0.0001

Mother takes the following supplements^1^						

… Folic Acid	84.2%	96.7%	77.7%	80.0%	83.7%	0.0111

… Iron	41.7%	93.6%	35.5%	57.1%	51.6%	<0.0001

… Ferrous Sulfate	79.3%	83.9%	76.0%	62.5%	77.4%	0.5889

… Any above supplements	90.5%	96.9%	86.7%	91.7%	90.1%	0.1537


Health posts by zone: Zone 1-Mazuko, Primavera Baja, Santa Rosa; Zone 2-Laberinto, Alto Libertad; Zone 3-El Triunfo, Nuevo Milenio, Jorge Chavez, La Joya, Pueblo Viejo; Hospital Santa Rosa; Zone 4-Hospital San Martin de Porres (Iberia). ^1^ Maternal residence and nutritional supplements have the following sample sizes in each zone: 39, 59, 140, and 12 for zones 1–4, respectively.

### 4. Data Management and Analysis

Surveys and biomarkers were stored in a secure office (and freezer) in the *Instituto de Investigaciones de la Amazonia Peruana* (IIAP) near Puerto Maldonado prior to shipment to our laboratory at Duke University. For each woman, we created a unique ID consisting of the two-digit year of enrollment, two-digit initials of the enrolling health post, two-digit month of enrollment, and two-digit number representing the n-th mother enrolled during that month. This code will identify the maternal family, with the mother assigned the letter A, newborn B, spouse/partner C and so on. All biomarkers (e.g., hair, blood, placenta, etc.) are recorded with the name of the participant and their unique ID.

A secure ACCESS database was created for enrollment, birth and follow-up survey data. 20% of surveys were screened for data entry errors and if error detection exceeded 10%, all surveys were screened. ACCESS data at enrollment and birth were exported and variables recoded for future analysis in SAS V9.4, R or STATA.

Trace element analyses were performed on hair and blood samples in the Hsu-Kim laboratory at Duke University using similar protocols are previously described [15,33,42]. Briefly, the proximal 2-cm length of hair was cut, weighed, and analyzed for total Hg content by thermal decomposition, amalgamation, atomic absorption spectrometry (Milestone DMA-80). The instrument was calibrated using diluted mixtures of a certified standard solution of 1 mg L^–1^ Hg^2+^ dissolved in 5% HNO_3_ (Brooks Rand). At the start of each batch run, the instrument calibration was confirmed with analysis of a standard reference material (SRM) hair samples (ERM DB001 Hg: 0.365 mg/kg) and verification of measured value within the certified concentration value. Analyses of analytical blanks and the SRM as repeated every 10 samples to verify instrument performance.

The concentrations of Hg and other trace elements (Cr, Mn, Fe, Cu, Zn, As, Se, Cd, Pb) were quantified in whole blood samples by inductively coupled plasma mass spectrometry (ICP-MS) (Agilent Technologies 7900). First, a 0.5 mL aliquot of a thawed blood sample was transferred to pre-cleaned polypropylene test tubes and digested with 1 mL of ultra-trace clean nitric acid (70%, SCP Science) and 0.05 ml of ultra-trace clean hydrochloric acid (35%, SCP science) for two hours at 65°C. After two hours, the samples were allowed to cool and 1 ml of ultra-trace clean hydrogen peroxide was added (30%, SCP Science) to the mixture. The blood was digested for an additional hour. After the digestions, the samples were cooled and spiked with 10 microliters of a 4 mg L^–1^ gold solution preserved in 5% HCl. Prior to ICP-MS analysis, the samples were diluted with a 2% HNO_3_/0.5% HCl (v/v) diluent. The diluent included internal standards at a 20 µg L^–1^ concentration (^45^Sc, ^89^Y, ^103^Rh, ^115^In, ^159^Tb, ^193^Ir, ^197^Au, and ^209^Bi). All elements were analyzed in helium reaction gas mode, except for Se and Fe which were analyzed in hydrogen mode. An aliquot of caprine blood SRM (NIST SRM933c: levels 2, 3, or 4) was analyzed in parallel with each sample batch of 24. Additionally, for each batch of 24 samples, two blood samples were selected and digested in triplicate. The ICP-MS instrument was calibrated and tuned daily at the start of each batch run. Calibrations were verified by an aqueous SRM (High Purity Standards: CRM-TMDW-A). Instrument blanks and calibration checks were performed at least once per 30 samples in the batch run.

Results from metals analysis will be compared against measures of newborn development, including APGAR score, anemia (measured clinically at birth and every post-natal follow-up), birth weight and size, two-year growth (clinically measured at each post-natal follow-up), and cognitive development (via the ASQ-3).

## III. Findings to Date, Characteristics of Women Followed at Birth

Of the 270 pregnant mothers enrolled, we successfully followed up 215 (80%) mothers at childbirth (***Table 2***). One of these 215 mothers had twins and an additional enrolled mother had a stillbirth. Among these women, we obtained 214 maternal and cord blood samples, 211 maternal and 212 infant hair samples, 212 placenta samples, 210 saliva samples, and 214 DBS. Lab processing of trace element contents for blood samples are complete; analysis of hair samples is ongoing. All other samples are stored for future use in a -80C freezer at Duke.

**Table 2:** Characteristics of Mothers Followed at Birth.


VARIABLE	ZONE OF ENROLLMENT				ALL ZONES	P-VALUE, DIFFERENCE BY ZONE

	1	2	3	4		

N (%)	37 (17%)	50 (23%)	121 (56%)	7 (3%)	215	

Median Age, years (mean)	27 (27.2)	27.5 (27.8)	28 (27.7)	23 (23.9)	28 (27.6)	0.1033

Hemoglobin (Hb) Level^2^						

… Moderate Anemia (Hb 7.0–9.9)	5.4%	4.2%	9.2%	0.0%	7.1%	0.9041

… Anemia (Hb 10.0-11.9)	62.2%	66.7%	61.7%	66.7%	63.0%	

… Normal (Hb ≥ 12)	32.4%	29.2%	29.2%	33.3%	29.9%	

Short Stature, % ht ≤ 1.45m^3^	11.1%	22.0%	6.7%	20.0%	11.4%	0.0350

Total Prenatal Visits	7.5 (2.1)	7.9 (1.8)	6.9 (2.2)	7.4 (2.4)	7.2 (2.10)	0.0253

Hypertension during pregnancy, percent^4^	8.3%	8.2%	5.2%	0.0%	6.3%	0.7428

Emergency Cesarean^5^	19.4%	8.0%	11.6%	0.0%	11.7%	0.2973

Birth attendant ….. Physician	18.9%	20.0%	17.4%	28.6%	18.6%	0.7858

…. Obstetrician	78.4%	78.0%	77.7%	57.2%	77.2%	


Health posts by zone: Zone 1-Mazuko, Primavera Baja, Santa Rosa; Zone 2-Laberinto, Alto Libertad; Zone 3-El Triunfo, Nuevo Milenio, Jorge Chavez, La Joya, Pueblo Viejo; Hospital Santa Rosa; Zone 4-Hospital San Martin de Porres (Iberia). ^2^ No women were classified as having severe anemia (Hb < 7.0). Hb level reported is from the last prenatal visit, sample sizes by zone are: 37, 48, 120, 6. ^3^ Short Stature sample sizes by zone are: 36, 50, 120, and 5. ^4^ Hypertension during pregnancy sample sizes by zone are: 36, 49, 115, 7. ^5^ Emergency cesarean has a sample size in zone 1 of 36.

Median age of women was 28 years, similar to enrollment. Malnutrition prevalence was high, with 70% of women classified as anemic at the time of childbirth (hemoglobin under 12 g/dL) and 11% of short stature. Anemia was high across all zones, while short stature was more prevalent in zones 2 and 4. The total number of prenatal care visits was highest in zone 2 (7.9 visits) and lowest in zone 3 (6.9 visits). Hypertension prevalence during pregnancy was low overall (6% of pregnancies) but exceeded 8% in zones 1 and 2. Overall prevalence of cesarean births was 18%, but 11.7% were emergency cesareans and occurred primarily in zone 1.

There were 55 women lost to birth follow-up. Of these, 28 (51%) were lost due to their failure to notify the study team when the birth was occurring. Another 14 (25%) gave birth in another location (Cusco, Lima, etc.). The remaining lost to follow-up include two mothers who had an abortion and 11 with variable reasons such as lacking cell phones to call the study team, nervous about the study, or had a rapid labor such that they delivered at home or somewhere outside the health post. These women lost to follow-up did not significantly differ from those followed up by maternal age, zone, gestational age at enrollment, or stature. However, women not followed at birth had significantly fewer prior pregnancies compared to those who were followed (2.6 vs. 3.3, p = 0.0013). All women are being re-contacted for follow-up regardless of whether birth data were collected.

Characteristics of the newborn are important indicators for impacts of in-utero exposure as well as indicators of early development during the first two years of life. We use several indicators to determine fetal growth outcomes. First, we use World Health Organization definitions for preterm births in three categories based on gestational age (Extremely preterm, <28 weeks; Very preterm, 28 to <32 weeks; Late preterm, 32 to <37 weeks) [55]. Second, in the clinic, health care professionals estimated small for gestational size using standard protocols. Finally, we use the standard cutoff of 2500 g at birth as low birthweight. Additional measurements at birth include 1-minute and 5-minute APGAR scores.

Of the 216 children enrolled, 55.6% were male, which was relatively consistent across zones (***Table 3***). The average gestational age was 39 weeks, with only 5% of births occurring preterm. Similarly, 5% of children were classified as small-for-gestational age and 2% were born under 2500 g. APGAR scores were normal for almost all children. A surprising result was the large size of newborns: 35% were considered large gestational age and 56% were over 3500 g at birth. None of these birth outcomes varied by zone of enrollment (***Table 3***).

**Table 3:** Characteristics of Newborns.


VARIABLE	ZONE OF ENROLLMENT				ALL ZONES	P-VALUE, DIFFERENCE BY ZONE

	1	2	3	4		

N (%)	37 (17%)	51 (24%)	121 (56%)	7 (3%)	216	

Male, %	51.1%	68.3%	51.2%	57.1%	55.6%	0.194

Gestational age at birth, weeks (SD)^1^	39.1 (2.13)	39.3 (1.41)	38.8 (3.40)	39.0 (1.87)	39.0 (2.82)	0.7613

Gestation: % born before 37 weeks^1^	2.8%	8.5%	4.1%	11.1%	5.3%	0.4575

Size for gestational age (clinical)						0.4072

…. Small	2.7%	3.9%	5.8%	0.0%	4.6%	

…. Large	32.40%	35.30%	38.00%	0	35.20%	

Birth weight^2^						0.1677

…. <2500 g	0.0%	2.0%	2.5%	0.0%	1.9%	

…. 2500–3000	13.9%	5.9%	9.1%	9.0%	8.8%	

…. 3000–3500	36.1%	27.5%	31.4%	85.7%	33.0%	

…. >3500	50.0%	64.7%	67.0%	14.3%	56.3%	

APGAR < 7, 1 minute^2^	0.0%	3.9%	2.5%	0.0%	2.3%	0.6575

APGAR < 7, 5 minutes	0.0%	2.0%	1.0%	0.0%	1.0%	0.8000


Health posts by zone: Zone 1-Mazuko, Primavera Baja, Santa Rosa; Zone 2-Laberinto, Alto Libertad; Zone 3-El Triunfo, Nuevo Milenio, Jorge Chavez, La Joya, Pueblo Viejo; Hospital Santa Rosa; Zone 4-Hospital San Martin de Porres (Iberia). ^1^ Gestational age at birth has the following sample sizes, respectively by zone: 36, 46, 120, 7. ^2^ Birthweight and APGAR score sample sizes by zone: 36, 47, 120, 7.

## IV. Strengths and Limitations

This unique birth cohort measures in-utero metals exposure of mother-child dyads in an ASGM region where there is a high prevalence of anemia and malnutrition, yet low prevalence of low birthweight and small for gestational age. In other seminal birth cohorts, sample sizes are larger, and they are typically conducted in developed or urbanized regions where women have access to high quality prenatal care. The high prevalence of malnutrition in our sample allows for the evaluation whether these disorders alter the toxicological paradigm for defining levels of risk when such populations are exposed to toxic trace metals, including risk for developmental toxicity.

In addition, our unique sample benefits from high motivation of mothers to remain connected to the study, aiding our ability to conduct the 36–48 month follow-up and enables construction of risk profiles for infants born to WCBA living in ASGM regions. Thanks to our community and individual engagement, mothers remain steadfast in their support with less than 1% of mothers communicating any reservations about participation. This support has resulted in our ability to maximize biomarker collection even for samples that mothers know they may not learn of results for years to come due to funding constraints.

Another major strength of this study is the extensive collection of biomarkers (toxicological exposure assessment), clinical and health outcomes (growth, cognition, epigenetics), and socio-demographic data that enable cross-cutting analyses of environmental factors impacting human health. This is of primary interest to local and regional health and environmental agencies, with whom we have fostered a strong partnership. DIRESA- Madre de Dios has provided access to clinical records as well as helped inform physicians and nurses of our study. The high rate of post-natal visitation has also enabled the use of in-depth clinical data on the children regarding vaccination dates, anthropometry, illnesses, medications, and nutritional supplements, among others.

One potential limitation of this study is the relatively small sample size compared to birth cohorts in the Seychelles and Faroe Islands. However, as noted earlier, our enrollment sample of 270 mothers represents between 4.4% and 7% of all births in the region. In addition, our sample size of 215 mother-child dyads still allows us to test our major hypotheses and it is possible for us to augment the sample using the same protocols if additional data are needed for future tests. Another potential limitation in our study is the low prevalence of low birthweight (2%) and small for gestational age (5%) compared to 4.3% and 1.2% reported from ENDES, respectively. It is possible that, given our engagement with mothers, risk for low birthweight decreased, but we expect differences in our clinical definition of these outcomes vs. maternal recall in ENDES.

Another limitation of our study is the delay in the original 24-month follow-up due to COVID-19. The study design will remain unchanged when we restart; however, we may suffer from loss of participants due to migration or death.

## V. Collaboration

We welcome collaborations with other researchers. We have a collaborative research team consisting of experts in environmental science, toxicology, epidemiology, biostatistics, and clinical research. Researchers interested in collaborating can visit our website for more information (*https://sites.globalhealth.duke.edu/panlab/*) and email the PI, William Pan (*William.pan@duke.edu*).

## Additional File

The additional file for this article can be found as follows:

10.5334/aogh.3152.s1 Supplemental Figure 1. CONAMAD Mother’s Date of Enrollment and Expected Date of Birth at the time of enrollment.
