# Supplementary figures and images for: CoNaMad—Cohorte de Nacimiento de Madre de Dios/Madre de Dios Birth Cohort to Study Effects of in-utero Trace Metals Exposure in the Southern Peruvian Amazon
Source: Ann Glob Health. 2021 Jul 19;87(1):69. doi: 10.5334/aogh.3152 (PMC8300581; doi:10.5334/aogh.3152)

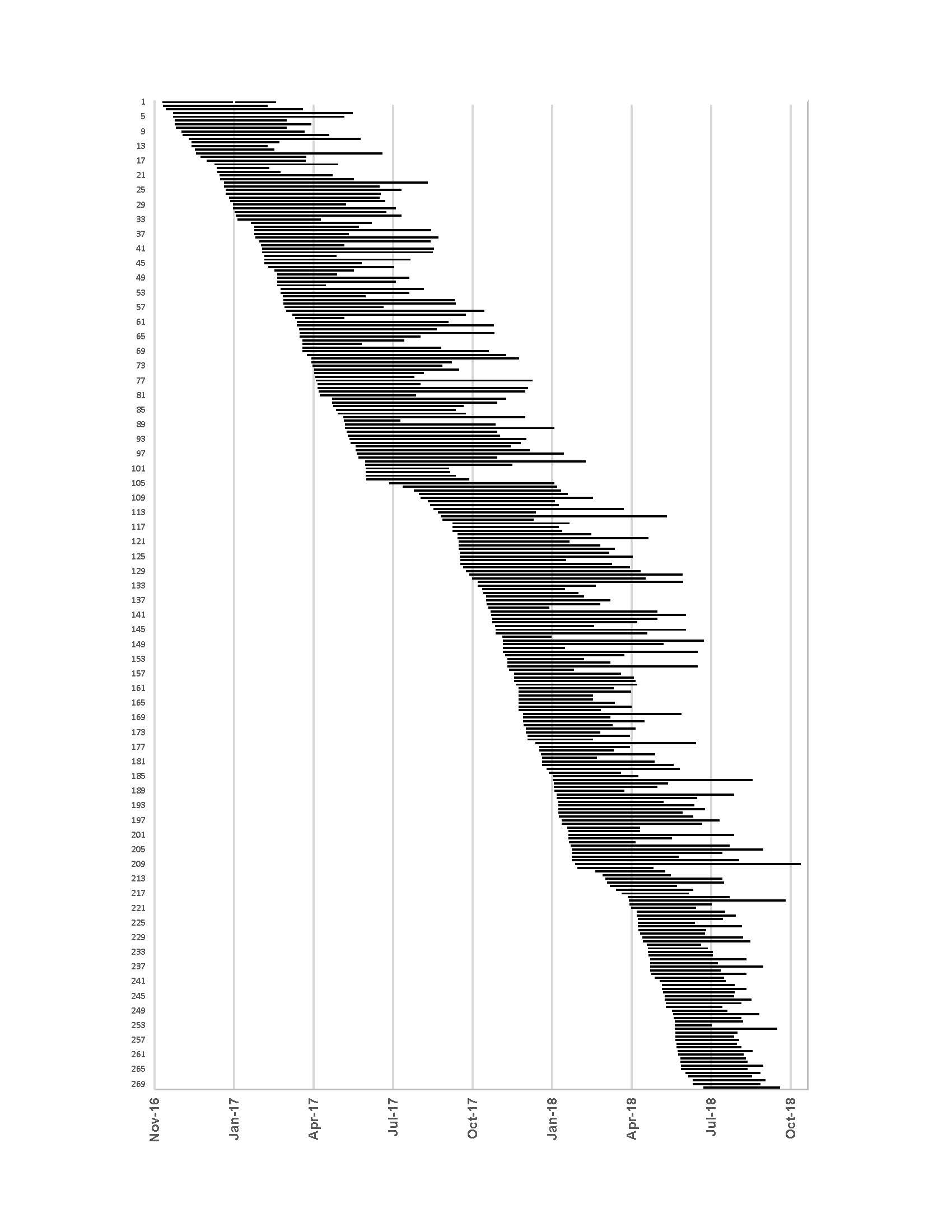

Supplement: Supplemental Figure 1. — CONAMAD Mother’s Date of Enrollment and Expected Date of Birth at the time of enrollment. [file agh-87-1-3152-s1.jpg]
